# Novel ^1^H low field nuclear magnetic resonance applications for the field of biodiesel

**DOI:** 10.1186/1754-6834-6-55

**Published:** 2013-04-16

**Authors:** Paula Berman, Adi Leshem, Oren Etziony, Ofer Levi, Yisrael Parmet, Michael Saunders, Zeev Wiesman

**Affiliations:** 1 The Phyto-Lipid Biotechnology Lab, Departments of Biotechnology, Energy and Environmental Engineering, Ben-Gurion University of the Negev, P.O. Box 653, Beer-Sheva, 84105, Israel; 2 Department of Industrial Engineering and Management, Ben-Gurion University of the Negev, P.O. Box 653, Beer-Sheva, 84105, Israel; 3 Department of Management Science and Engineering, Stanford University, Stanford, CA, USA

**Keywords:** ^1^H low field nuclear magnetic resonance, Biodiesel, Biodiesel physical properties, Chemometrics, Laplace inversion, Transesterification

## Abstract

**Background:**

Biodiesel production has increased dramatically over the last decade, raising the need for new rapid and non-destructive analytical tools and technologies. ^1^H Low Field Nuclear Magnetic Resonance (LF-NMR) applications, which offer great potential to the field of biodiesel, have been developed by the Phyto Lipid Biotechnology Lab research team in the last few years.

**Results:**

Supervised and un-supervised chemometric tools are suggested for screening new alternative biodiesel feedstocks according to oil content and viscosity. The tools allowed assignment into viscosity groups of biodiesel-petrodiesel samples whose viscosity is unknown, and uncovered biodiesel samples that have residues of unreacted acylglycerol and/or methanol, and poorly separated and cleaned glycerol and water. In the case of composite materials, relaxation time distribution, and cross-correlation methods were successfully applied to differentiate components. Continuous distributed methods were also applied to calculate the yield of the transesterification reaction, and thus monitor the progress of the common and in-situ transesterification reactions, offering a tool for optimization of reaction parameters.

**Conclusions:**

Comprehensive applied tools are detailed for the characterization of new alternative biodiesel resources in their whole conformation, monitoring of the biodiesel transesterification reaction, and quality evaluation of the final product, using a non-invasive and non-destructive technology that is new to the biodiesel research area. A new integrated computational-experimental approach for analysis of ^1^H LF-NMR relaxometry data is also presented, suggesting improved solution stability and peak resolution.

## Background

Biodiesel is defined as mono-alkyl esters of long chain Fatty Acids (FAs), offering a viable alternative to petroleum-based diesel fuel. Biodiesel has become more attractive recently because of diminishing petroleum reserves and the environmental consequences of exhaust gases from petroleum-fueled engines. It is simple to use, biodegradable, nontoxic and essentially free of sulfur and aromatics. Since it can be manufactured using existing industrial production capacity, and used with conventional equipment, it provides substantial opportunity for immediately addressing energy security issues [1]. As a consequence, biodiesel production grew in the last decade from 0.8 to 14.7 × 10^9^ l [2].

Biodiesel is commonly produced by a chemical reaction named transesterification (TE), where a lipid is reacted with an alcohol in the presence of a catalyst. It can be derived from a wide range of lipid-containing materials, although more than 95% is currently produced from edible-grade oils [3]. Large-scale production of biodiesel from edible resources can lead to imbalance in the global food market. Thus, alternative high yield and high quality feedstocks are continuously researched. These include non-edible oilseeds such as castor [4,5] and jatropha [6]; algae [7]; and waste materials such as recycled oils [8], municipal [9,10] and winery [11] wastes.

The quality of the biodiesel produced is of paramount importance to its successful commercialization [12]. Its performance in a compression-ignition engine is determined by the physical properties of the fuel attributed by the biodiesel composition, which corresponds to the FA profile of the parent lipid [13,14]. Severe operational problems including engine deposits, filter clogging, and fuel deterioration can be caused by residues of unreacted lipids such as sterols, acylglycerols, phospholipids, and Free Fatty Acids (FFAs), or residues such as glycerol, alcohols, and water in the biodiesel [12]. Therefore, to maintain proper vehicle performance, official international standards were established that require analyses consisting of chromatographic, spectroscopic, physical properties-based, and wet chemical methods. These methods are destructive, time consuming, laborious and environmentally unfriendly.

^1^H Low Field Nuclear Magnetic Resonance (LF-NMR) is a rapid non-destructive technology extensively used in the food, polymer, petroleum and pharmaceutical industries. It is widely used in industrial quality control for the determination of solid-to-liquid and oil-to-water ratios in materials as diverse as oil-bearing rock, food emulsions and plant seeds [15].

The field of ^1^H LF-NMR relaxometry is a powerful tool for identifying molecular species and to study their dynamics even in complex materials. This relates to the measurement of relaxation constants as a consequence of interactions among nuclear spins and between them and their surroundings. Longitudinal magnetization returns to equilibrium following application of a radio frequency field because of energy transferred to the lattice, and transverse relaxation arises from spin-spin interactions following a 90° pulse. The time constants for longitudinal and transverse relaxations are *T*_*1*_ and *T*_*2*_ respectively.

Relaxation time distribution experiments range from simple and rapid one dimensional (1D) tests to more complicated multidimensional ones. 1D tests use constant intervals between pulses, allowing for either longitudinal or transverse relaxation to be evaluated, whereas in multidimensional experiments, the signal is measured as a function of two or more independent variables, allowing the spin system to evolve under different relaxation mechanisms [16]. In biological samples, spins exist in a variety of different environments, giving rise to a distribution of relaxation times in which the measured relaxation decay is a sum of contributions from all spins [17].

Most commonly applied 1D tools are based on either acquisition of the free induction decay signal following a 90° pulse, or pulse sequences such as the spin echo [18], pulsed field gradient spin echo [19], CPMG [20,21] or inversion/saturation recovery [22]. Only few of the applications found in the literature, mainly for the food industry, include measuring oil content in low moisture oilseeds [23-25], algae [26], and meat [27]; for solid fat content measurement [28]; water holding capacity in meat and fish, [29-31]; characterization of water in agro-food products [32]; molecular mobility in wheat starch [33]; study of the denaturation of proteins in eggs and whey [34]; effect of formulation on liquid and solid fat ice cream [35]; prediction of viscosity, cetane number, and iodine value of oilseeds [36]; drug delivery [37]; and many others.

More recently, new two-dimensional (2D) relaxation time distribution pulse sequences have been suggested, including *T*_*1*_*T*_*2*_[16], *T*_*2*_-store-*T*_*2*_[38] and *T*_*2*_-D [39]. Several of the applications published in the last decade include 2D relaxation/diffusion correlations in porous media [39-41]; determination of avocado maturity [42]; monitoring the effect of high pressure and microwave processing on the microscopic water distribution and starch chain dynamics in potato and starch [43]; investigation of the physiological changes associated with ripening and mealiness in apples [44]; peak assignment to cell components, including compartmentalized water, pectins, starch, protein, and hemicelluloses in carrots [45]; and peak assignment for exploratory purposes in other foodstuffs including eggs, fish, dairy products, salad cream, and cake [46].

The speed with which data is obtained and the complexity of the signal acquired can become overwhelming unless suitable methods for interpretation are used. Data analysis of relaxation time distribution experiments is traditionally performed in one of several ways:

a. By projecting the data into new coordinates that maximize the original variance. This can be applied only on a group of observations, as the model looks for commonalities in the original data. The main advantage is that the method imposes no mathematical constraints. This field is termed chemometrics and it comprises the application of multivariate statistics, mathematics, and computational methods to chemical measurements to enhance the productivity of chemical experimentation [47]. Chemometric methods include clustering techniques (to spot differences between samples, detection of outliers, and grouping) and regression models (for correlating NMR measurements to reference data) [48].

b. By assuming discrete multi-exponential behavior of the data. Coefficients are extracted using nonlinear fitting models, and the coefficients can be used in prediction models [49-52].

c. By assuming a continuous distribution of exponentials. Here a relaxation time distribution of exponential coefficients is achieved with components appearing as peaks. This is an ill-posed Inverse Laplace Transform (ILT) problem. The common mathematical solution implemented today, for both 1D and 2D data, is based on *L*_*2*_-norm regularization [16,17,53-55].

The goal of this summary is to show the potential of applying ^1^H LF-NMR technology to the field of biodiesel. We detail novel applications based on 1D (CPMG) and 2D (*T*_*1*_*T*_*2*_) pulse sequences for (a) screening new alternative biodiesel resources in their whole conformation, (b) monitoring the biodiesel TE reaction, and (c) evaluating the quality of the final product. A new algorithm for the 1D ILT problem, suggested by our research team, is also presented. This new approach applies *L*_*1*_-norm regularization to find sparse solutions, using a formulation suitable for the PDCO solver (Primal-Dual interior method for Convex Objectives) [56]. In comparison, the common *L*_*2*_-norm (least squares) result has been found to contribute to the broadening of peaks.

## Results and discussion

**Figure 1:**
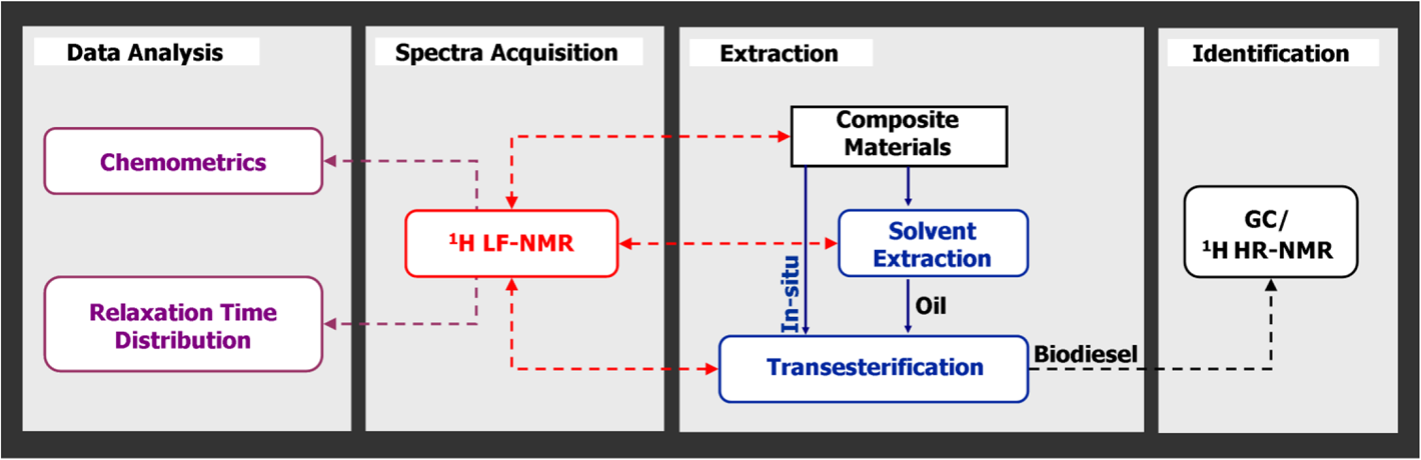
**Schematic representation of work methodology.** Work methodology consisted of four steps: Extraction of material, signal acquisition using ^1^H LF-NMR, identification of constituents using standard chromatographic and spectroscopic methods, and finally applying different data analysis methods to correlate the acquired information.

The methodology for performing the research presented hereby is detailed in Figure 1. This multidisciplinary research consists of applying different data analysis methods to correlate the information acquired with ^1^H LF-NMR on different materials to standard chromatographic and spectroscopic methods.

### Selection of alternative biodiesel feedstocks

To successfully market a new biodiesel feedstock, the biodiesel overall production process should be cost-effective to compete with petrodiesel prices. Zhang et al. [57] reported that 70–95% of the biodiesel production cost is the price of the feedstock itself. Therefore, high oil content sources are favored. In addition, biodiesel must meet international quality standards, several of which are related to the FA composition of the parent oil, thus determined by choice of feedstock. Hence, oil content and physical properties related to FA composition are important parameters in the successful commercialization of a new feedstock.

Oil content has long been measured using ^1^H LF-NMR. In addition, biodiesel quality parameters were successfully predicted from oilseeds relaxation signals [36]. The main component in dried oilseeds is the oil constituent; therefore the acquired ^1^H LF-NMR signal can be directly related to oil quantity and quality. Different composite materials consisting of additional components such as fibers, compartmentalized water and others require a more comprehensive analysis of the relaxation signals using ILT analysis.

In the following section, we present two ^1^H LF-NMR tools that can be applied for finding new alternative biodiesel feedstocks. The first involves simultaneous screening of oilseeds by viscosity and oil content using chemometric tools. The second involves assigning peaks to the different components in olives using 1D and 2D tools, and thus relates only to the desired components.

#### Simultaneous screening of oilseeds by viscosity and oil content using chemometrics

The standard relaxation time distribution method, for oil content measurement in oilseeds, consists of acquisition of a single intensity signal and its correlation to oil content, to construct a calibration curve according to the international standard [58]. Each type of oilseed requires a specific calibration curve, since different types of oilseed hold unique FA profiles and therefore result in unique slopes and intercepts. This procedure is relatively simple but has several weaknesses, including (a) loss of qualitative information, (b) lack of robustness, since univariate models cannot properly handle outliers that result from abnormal signals or varied quality of oilseeds, and (c) applicability only to oilseeds whose calibration curve has already been established.

Screening of suitable biodiesel feedstocks often involves sampling extremely large batches of samples. Here a new protocol is suggested for qualitative and quantitative large-scale screening of oilseeds. A fast application that provides comprehensive information on observations will simplify the characterization and quantification of new and existing alternative biodiesel resources. This is very important to this field.

The protocol was demonstrated using the acquired 1D transverse relaxation data of nine oils, each approximately 1, 2, 3, and 4 ml, that were correlated against oil content. The different oil volumes were used to represent varying oil contents. Oils were chosen for the application because of the possibility of controlling oil content with constant FA content of each sample from the same oil source. Unsupervised data exploration was initially conducted using Principal Component Analysis (PCA). The oils used in this analysis are almond (ALM), canola (CAN), castor (CAS), linseed (LIN), mustard1 (MUS-A), mustard2 (MUS-B), olive (OLI), soy (SOY) and sunflower (SUN).

**Figure 2:**
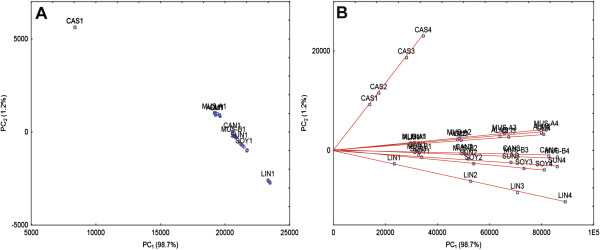
**Simultaneous screening of oils by viscosity and oil content using chemometrics.** Oils were chosen for the application because of the possibility of controlling oil content with constant FA content of each sample from the same oil source. PCA score scatter plots of *PC*_*1*_ and *PC*_*2*_ are shown for: (**A**) The covariance matrice pre-processed by the 1st intensity value. Since all the samples per type of oil were very closely clustered, the name of only one of the oil samples, for each of the oils, is shown. (**B**) The covariance matrice without pre-processing. Fitting of the calculated *PC*_*1*_ and *PC*_*2*_ for each of the individual oils separately yielded a perfect linear regression (the curves for each of the separate oils are illustrated on the plot).

PCA score scatter plots of the first two Principal Components (*PC*_*1*_ and *PC*_*2*_) extracted using the covariance matrice pre-processed by the first value or without pre-processing, are described in Figure 2A and 2B. Figure 2A shows no information related to oil content, given that almost no differentiation was observable between the different volumes from the same type of oil. Since all the samples per type of oil were very closely clustered, the name of only one of the oil samples for each of the oils is shown on the plot, to facilitate its interpretation. Still, *PC*_*2*_ revealed qualitative information, meaning high or low values of *PC*_*2*_ correlated to high or low viscosities, as shown in a previous study [59]. In that study, a PCA model was suggested for rapid large-scale screening of castor seeds according to viscosity. An excellent correlation was found there between viscosity and *PC*_*2*_ calculated from the 1D transverse relaxation data, acquired on castor genotypes.

In Figure 2B on the other hand, both *PC*_*1*_ and *PC*_*2*_ held significant information regarding oil content. Fitting of the calculated *PC*_*1*_ and *PC*_*2*_ for each of the individual oils separately yielded an excellent linear regression (without intercept). The slope extracted from each of these curves was found to linearly correlate with the ln(*T*_*2*_) of the oils (y = -0.49ln(*T*_*2*_) + 2.54, R^2^=0.99; the Figure was added in an additional file [see Additional file 1]). *T*_2_ was the average value for each of the 4 samples per oil, calculated using monoexponential fitting.

Based on the unsupervised results shown, it was assumed that construction of an oil content (according to the oil weight) calibration including various types of oils would be possible using multivariate regression. Partial Least Squares (PLS) was then applied on the unprocessed covariance matrice. Training of the model was performed using three samples per oil, and the fourth was used for validation. The established model showed excellent prediction capability, as calculated on the validation set (R_P_^2^=0.99, Root Mean Squared Error of Prediction (RMSEP) = 0.075 g, the Figure was added in an additional file [see Additional file 2]). This suggests that the PLS model considers the decay rate of the specific oil, and generates an oil content reading accordingly. This model met the initial goal of eliminating the need for constructing specific calibration curves for each oil quality composition. These are important findings, suggesting that both quantitative and qualitative information can be extracted using PCA for data exploration and PLS for prediction.

#### Selection of alternative biodiesel feedstocks according to chemical composition

As a first step in characterizing lipid components in composite materials using ^1^H LF-NMR, for ultimately screening new feedstocks, we chose olives as a test case. This work was performed in collaboration with Dr. Brian Hills from the Institute of Food Research (Norwich, UK).

A major opportunity within the olive oil production industry is the exploitation of certain by-products obtained during the processing of olives for oil, such as pomace and olive pits, which can be used as biofuels [60]. Olive fruits have been studied for many years from an analytical point of view. The olive fruit is an ovoidal drupe consisting of an epicarp (1.5-3.5% of the fruit weight), mesocarp (or pulp, 65-83%), and kernel (or stone, 13-30% of fruit weight). Its average chemical composition is water 50%, oil 22%, proteins 1.6%, sugar 19.1%, cellulose 5.8% and ash 1.5% [61].

In this study, 1D and 2D pulse sequences were used for signal acquisition of olive oil, a whole fresh olive fruit, olive stone, and dry whole olive fruit samples. 1D relaxation time distribution using the WinDXP ILT software package, and 2D cross-correlation ILT software, were used for data analysis. Peaks were provisionally assigned to olive components by collating information from the different samples, and according to published peak assignment on avocado, apples, and xylem and phloem of carrots, as described below. Real peaks were marked A-H and cross peaks were marked according to the two exchanging components (e.g. BF stands for proton exchange of peaks B and F).

##### 1D relaxation time distribution

**Figure 3:**
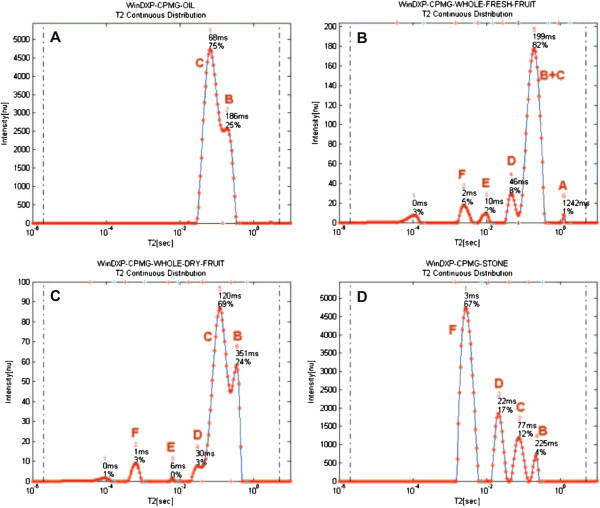
**Peaks assignment of the 1D relaxation time distributions of olive constituents.** Continuous distribution of exponentials of (**A**) olive oil, (**B**) whole fresh olive, (**C**) whole dry olive, and (**D**) olive stone were calculated using the WinDXP ILT software package. Peaks in each Figure were assigned (A-F) to olive components by collating information from the different samples, and according to published peak assignment on avocado, apples, and xylem and phloem of carrots.

The olive oil 1D peak distribution consists of peaks B and C (Figure 3A). Olive oil contains mostly triacylglycerols (98-99%); therefore, peaks B and C were assigned as oil peaks. These two oil peaks agree with the bi-exponential behavior of oil in oil-containing materials as previously suggested [25,50]. This result also coincided with previous peak assignment in avocado [42].

The 1D olive fruit sample contains four peaks in addition to the two oil peaks (Figure 3B). Interestingly, in the oil sample, peak C has a higher relative intensity than peak B, whereas in the fruit this ratio is flipped. As noted before, fresh olives consist of approximately 50% water. Since these components are the major peaks in the fruit sample, and based on previous peak assignment [44], it was assumed that they arise also from cytoplasmic and extracellular water. This was validated using the dry whole olive sample (Figure 3C).

Peak A in Figure 3B was assigned as vacuolar water because of the high fluidity of water molecules in this organelle as suggested for apples [44]. Indeed, in a dissected olive sample, peak A changed into one of the major peaks (data not shown), probably because of free water released as a consequence of dissecting the olive flesh. Peak E did not appear in the dissected olive flesh sample. Its relative intensity in a dry olive sample was significantly reduced compared to the fresh sample, suggesting that water had been evaporated, though not entirely. Peak E was therefore assigned as a water component in the olive stone. The chemical composition of olive stones (% dry weight) according to Heredia et al. [62] consists almost entirely of fibers (80%), around 10% moisture, 5% oil and some other minor components. Based on this information, it is reasonable to assume that the peaks in the olive stone sample, with the lowest relative importance, are the oil components B and C, whereas peak D probably arises from a water component (Figure 3D).

Peak F in apples was assigned as water associated with the rigid components of the cell wall [44]. This however did not coincide with the relative intensity of this peak in the stone sample (Figure 3D), where it is the principal component. As previously stated, the major constituent compatible with this peak, is the lignocellulosic material, which frameworks the cell wall, with hemicellulose, cellulose and lignin as the main components. MacKay et al. [63] suggested that the primary cell wall molecules (not including water) can be divided into a practically rigid fraction consisting of all the cellulose and some of the hemicellulose molecules; and a higher mobility fraction consisting of pectic polysaccharide and some hemicellulosic molecules. At this point, it is difficult to determine whether peak F arises from these very fast relaxing molecules, or from water attached to them, and will be further discussed using 2D cross-correlation measurements.

##### 2D cross-correlation

**Figure 4:**
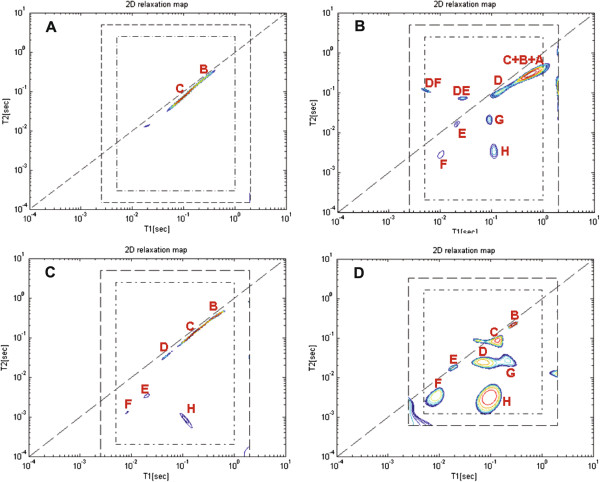
**Peaks assignment of the 2D cross correlation time distributions of olive constituents.***T*_*1*_*-T*_*2*_ correlation time distribution of (**A**) olive oil, (**B**) whole fresh olive, (**C**) whole dry olive, and (**D**) olive stone were calculated using an in-house 2D-ILT tool [15]. Peaks A-F on the diagonal consist of the high mobility molecules as previously assigned. The two new off-diagonal peaks (marked G and H) in the area where *T*_*2*_*<T*_*1*_ were assigned as the higher mobility hemicellulosic fraction (Peak G) and the more rigid cellulosic constituent, as they are the major peaks in the stone sample.

Figures 4A-4D show the 2D cross-correlation relaxation time distributions analyses for the olive oil, whole fresh and dry olive fruit, and olive stone samples respectively. The previously assigned peaks A-F appear here on the diagonal where *T*_*1*_≈*T*_*2*_. Here two new peaks (marked G and H) appear off-diagonal in the area where *T*_*2*_<*T*_*1*_. These were assigned as the higher mobility hemicellulosic fraction (Peak G) and the more rigid cellulosic constituent, as they are the major peaks in the stone sample (Figure 4D). This coincided with previous peak assignment in xylem and phloem of carrots [45]. A summary of the relaxation contribution assignment is presented in Table 1.

**Table 1:** Peaks assignment of olives according to relaxation time distribution and cross correlation experiments

**Description**	**Peak**
Vacuolar/free water	**A**
Oil 1 and cytoplasmic water	**B**
Oil 2 and extracellular water	**C**
Pectin and extracellular water	**D**
Water in fibers	**E**
Cell wall water	**F**
Hemicellulose (stone)	**G**
Cellulose (stone)	**H**

The advantage of using multidimensional cross-correlation methods is apparent from comparing Figures 3B and 4B. Components G and H have similar transverse relaxation times as peaks E and F, and therefore cannot be distinguished in a 1D representation. This is most emphasized for the stone sample as discussed before in the assignment of Peak F. In addition, cross-correlation methods can be used to study proton exchange, which is not discussed in this work.

Relaxation time distribution analysis and cross-correlation methods allow the identification of components in composite materials. These can be used to identify separately oil, fibers, compartmentalized water and more, to search for new biodiesel feedstocks. The application of these methods in combination with simple linear and/or multivariate regression methods, can lead to the construction of intelligent and robust calibration curves and prediction models. The construction of an oil content calibration curve of olive mill pomace, using relaxation time distribution analysis, was recently shown by our research team [64]. In this study only eight olive mill pomace samples were used. Full leave one out cross validation yielded R^2^=0.83.

### Monitoring of the biodiesel TE reaction

The conditions and materials of the TE reaction may vary significantly according to the type of lipid substrate. The most common TE reaction is performed under alkaline conditions by reacting oil with methanol, because of its lower price compared to other alcohols. The alkali catalyst leads to a relatively fast conversion, while requiring only a moderate temperature. However, high yields are only achieved when the starting material is essentially free of moisture and consists of a low FFA content (<3%). Alternatively, low grade substrates and/or non-acylglycerol lipids can be reacted using Brönsted acids. Yet, the reaction is usually carried out at higher temperatures and for longer periods [65].

Another common biodiesel production process is an in situ TE reaction, which differs from the conventional reaction in that the oil-bearing material contacts with acidified or alkalized alcohol directly, instead of reacting alcohol with the pre-extracted oil. That is, extraction and TE proceed in one step, the alcohol acting both as an extraction solvent and as an esterification reagent [66]. This is a complicated process with several reactions and procedures, where the outcome of each one can potentially influence the quality of the following one, and of the final product.

The reactants in the TE reaction include the lipid substrate and alcohol, which is usually added in excess to drive the process forward. The desired product consists of high purity Fatty Acid Methyl Esters (FAMEs). However, residual glycerol, acylglycerol constituents (tri-, di- and mono-glycerides), alcohol, catalyst, water and others can often be found at diverse concentrations in the product, according to the reaction process. Therefore, assessment of the conversion of oil to biodiesel is required for monitoring and control of the production process in order to meet international biodiesel standards.

**Figure 5:**
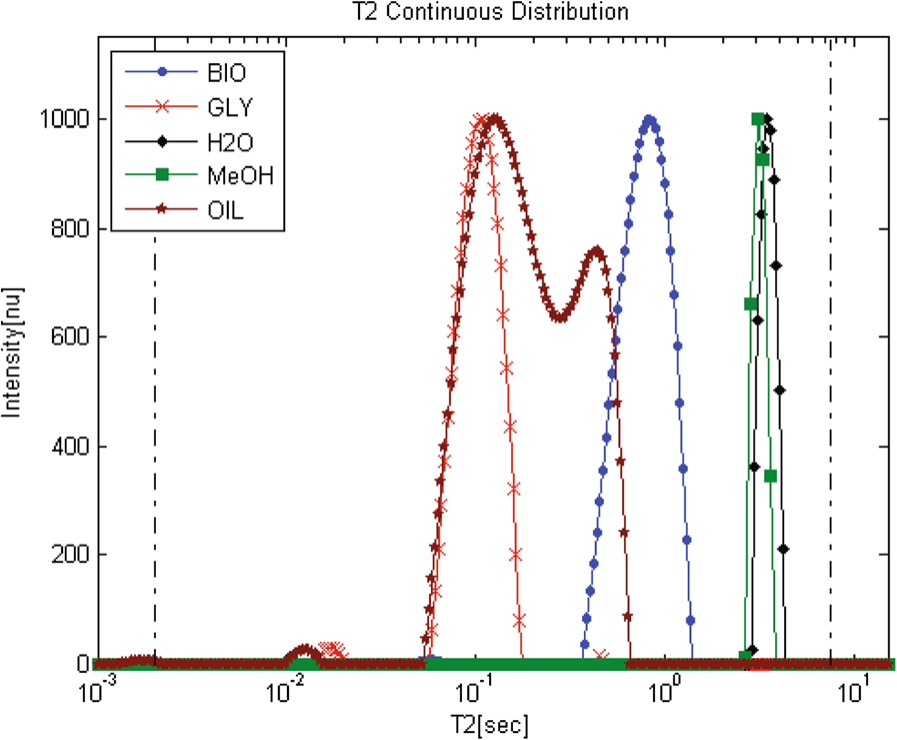
**WinDXP ILT analyses of biodiesel (BIO), glycerol (GLY), water (H2O), methanol (MeOH) and oil (OIL) samples.** Data acquisition and analysis were performed separately for each material. Relaxation time distributions were calculated using the WinDXP ILT software package**.** Each relaxation time distribution was normalized to its highest value for simplicity of comparison.

Monitoring of the TE reaction and biodiesel product using relaxation time distribution experiments, requires as a first step assigning peaks in the analyzed relaxation time distribution signals. Several of the components that participate in this reaction including soy oil, soy biodiesel, glycerol, water and methanol were sampled separately using ^1^H LF-NMR. Data was analyzed using the WinDXP ILT software package, and the combined relaxation time distributions are shown in Figure 5. Each solution was normalized to its highest value for simplicity of comparison. Here, the position of every component on the relaxation time distribution can be explained by its chemical structure. Water and methanol are polar, very mobile liquids, and thus have the largest *T*_*2*_ values. The biodiesel and oil samples consist of the same FA composition (biodiesel was produced from the analyzed oil). However, biodiesel consists of individual FAMEs (that have high freedom of movement and thus large *T*_*2*_) and oil, which consists mainly of triacylglycerols (three FAs esterified to a glycerol backbone) that have lower mobility and *T*_*2*_ because of this more rigid structure. Glycerol is a three-carbon molecule with three hydroxyl groups. As a result it has the lowest mobility and *T*_*2*_ because of hydrogen bonding.

**Figure 6:**
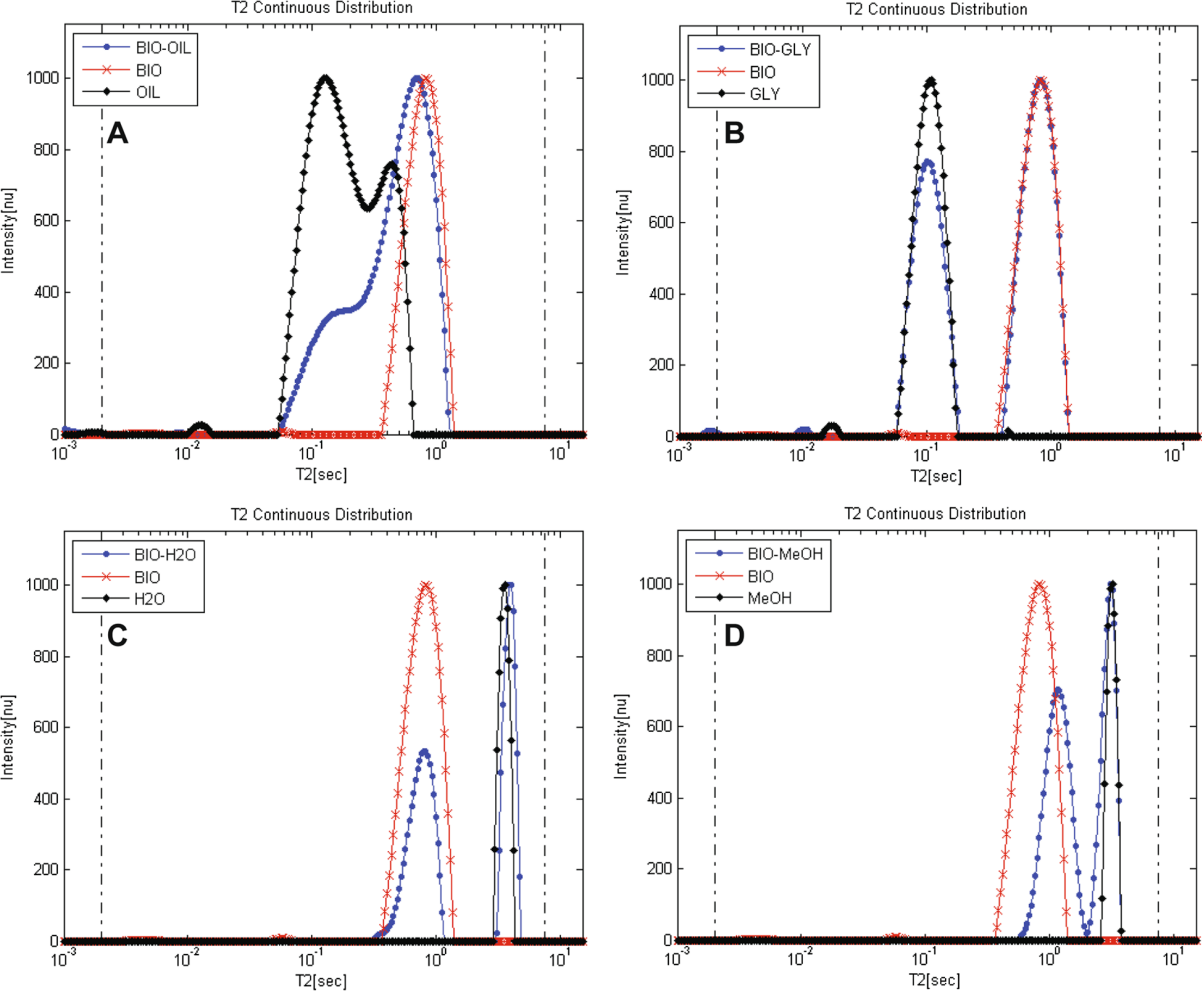
**1D continuous distribution of exponentials of biodiesel mixture samples.** Relaxation time distributions are shown for biodiesel and (**A**) oil (BIO-OIL), (**B**) glycerol (BIO-GLY), (**C**) water (BIO-H2O) and (**D**) methanol (BIO-MeOH) mixtures. Relaxation time distributions were calculated using the WinDXP ILT software package**.** The relaxation time distributions of the pure materials are shown for reference. The position of the different peaks in (**A**)-(**C**) is constant also for composite samples, though the peaks in the BIO-OIL mixture are difficult to resolve because of overlapping of components and widening of peaks imposed partly by the WinDXP ILT algorithm. In (**D**) the position of the biodiesel peak is shifted toward the methanol probably because of solubility interactions between these two components.

Figures 6A-6D show the analyzed relaxation time distributions acquired from mixtures (1:1 v/v) of biodiesel and oil, glycerol, water and methanol respectively (the relaxation time distributions of the pure materials presented above are shown for reference). As expected from their chemical composition, the biodiesel-water and biodiesel-glycerol mixtures formed two immiscible and separated phases inside the test tube, while the biodiesel-oil and biodiesel-methanol consisted of a single phase. As can be seen, the position of the different peaks in Figures 6A-6C is constant also for composite samples, though the peaks in the oil-biodiesel mixture are difficult to resolve because of overlapping of components and widening of peaks imposed partly by the WinDXP ILT algorithm. In the biodiesel-methanol mixture (Figure 6D), the position of the biodiesel peak is shifted toward the methanol component probably because of solubility interactions between the two components. This suggests that if methanol (or other alcohol) residues exist in the biodiesel; a shift is to be expected for the biodiesel peak in relation to its content.

**Figure 7:**
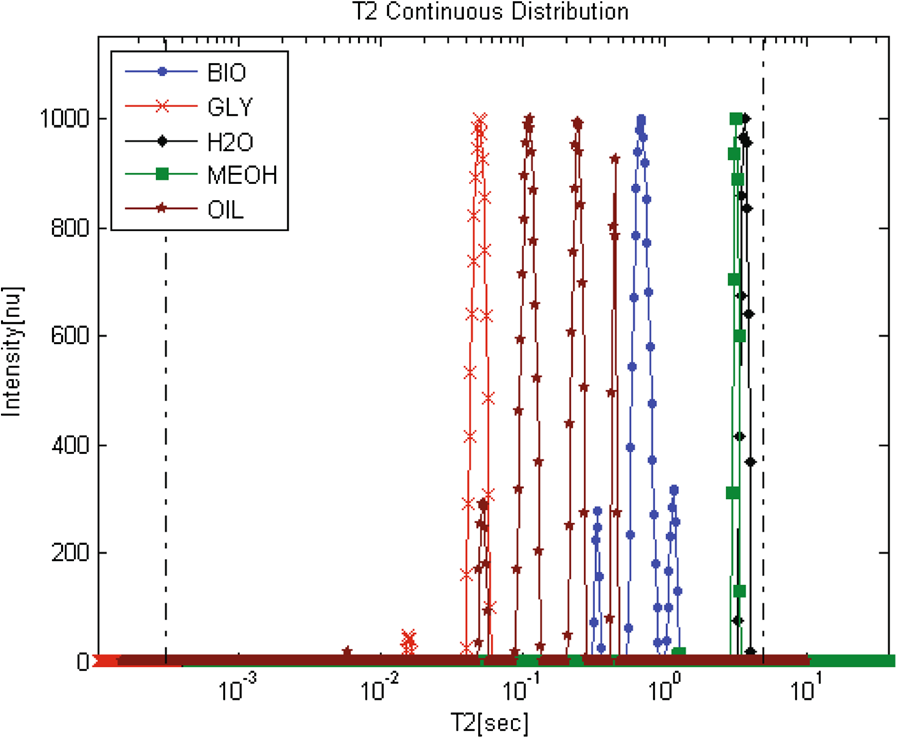
**PDCO analyses of biodiesel (BIO), glycerol (GLY), water (H2O), methanol (MeOH) and oil (OIL) samples.** This new algorithm shows improved resolution and sensitivity compared with the common *L*_*2*_ solution.

Application of the new *L*_*1*_-norm regularization based PDCO algorithm [56] to find sparse solutions (recently submitted article by our research group to Concepts in Magnetic Resonance A Journal) on the same biodiesel, glycerol, water, oil and methanol samples (Figure 7) showed better resolved relaxation time distributions and more accurate solutions. The PDCO analyzed relaxation time distributions of the oil and biodiesel samples reveal a larger number of peaks compared with the conventional results analyzed by the WinDXP ILT toolbox (Figure 5). As previously mentioned and as shown here, WinDXP analysis of oil reveals two overlapping peaks. Marigheto et al. [42] suggested that the peaks arise from molecules of differing mobility, such as the oleic and palmitic constituents, or from nonequivalent proton pools of different mobility, such as those on methyl and olefinic groups. In the current study, the PDCO algorithm reveals four resolved peaks instead of only two, suggesting promising unpublished information regarding identification of lipids constituents using relaxation time distribution experiments, and its supremacy as an analytical tool. Assignment of peaks to the appropriate components will be performed in the near future using standard materials.

To monitor the conversion reaction of oils to biodiesel, samples were acquired at different times during TE of rapeseed oil, and measured using relaxation time distribution experiments to track the progression of this reaction until completion. To calculate the yield of TE from the relaxation time distributions, a correlation with the data acquired by ^1^H High Resolution (HR)-NMR following the procedure of Meher et al. [65] was established. In situ TE process of olive mill waste was also monitored using relaxation time distribution experiments by measuring the relaxation signal of the pomace and biodiesel products following acid esterification, alkali TE and n-hexane extraction. Monitoring of this process led to successful optimization of reaction parameters.

#### Monitoring of the TE process and calculation of yield using ^1^H LF-NMR

As previously shown, oil and biodiesel mixtures can be evaluated using relaxation time distribution experiments. This can also be applied to monitor the progression of the TE reaction and to calculate its yield. Accurate measurement of the biodiesel yield, and analysis of the final FAME product for the presence of acylglycerol, is of paramount importance for establishing its quality.

**Figure 8:**
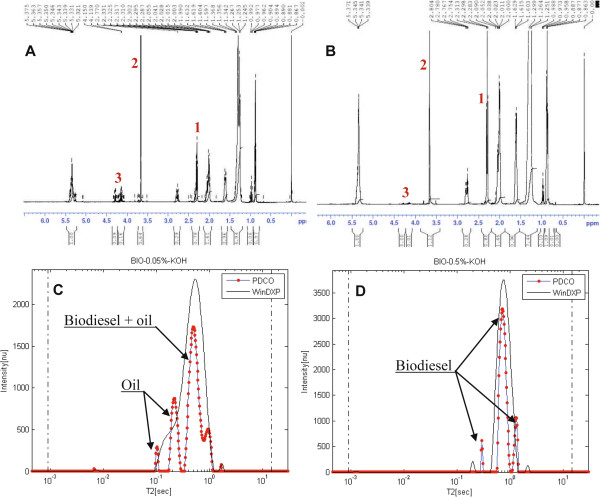
**Comparison of the NMR spectra and relaxation time distributions of two rapeseed biodiesel samples containing high and low acylglycerol residues.** Data was acquired using (**A**)-(**B**) ^1^H HR-NMR and (**C**)-(**D**) ^1^H LF-NMR of two rapeseed biodiesel samples A and B prepared with 0.05 and 0.5% KOH catalyst respectively, to achieve high and low acylglycerol residues. Peak numbers on (**A**) and (**B**) correspond to the peaks assignment in Table 2. Relaxation time distribution data was calculated using the WinDXP tool package and PDCO.

As a first step, two rapeseed oil biodiesels were prepared, each using 0.05% and 0.5% (w/w) KOH catalyst (samples A and B respectively). Yield of TE was calculated using ^1^H HR-NMR. Figures 8A and 8B show the 500 MHz ^1^H HR-NMR spectra acquired for samples A and B. The integrals of the peaks used for the yield calculations, as well as glyceryl peaks are listed in Table 2. Peak numbers are according to the numbers shown on the Figures. As expected from the low catalyst concentration, the integral of the glycerol peaks in sample A is larger, suggesting of high acylglycerol residues in the biodiesel. Consequently, samples A and B achieved yields of 54.7% and 93.3% respectively. Still, the last did not meet international standards (0.96% w/w total bound glycerol content, as measured from the same sample using Gas Chromatograph (GC), by an accredited European biodiesel laboratory, ASG Analytik-Service Gesellschaft mbH, Germany).

**Table 2:** **Peaks assignment and corresponding integrals of the**^**1**^**H HR-NMR spectra of samples A and B**

**Peak number**	**Functional group**^**a**^	**Chemical shift**^**a**^**[ppm]**	**Sample A**^**b**^**integral [nu]**	**Sample B**^**b**^**integral [nu]**
1	-OCO-C**H**_2_- (Acyl group)	2.23-2.36	0.78	0.80
2	R-OCO-C**H**_3_ (Methyl ester group)	3.7	0.64	1.12
3	-C**H**_2_OCOR (Glycerol group)	4.1-4.32	0.25	0.01

^a^ Peaks were assigned according to Meher et al. [65] and Guillén and Ruiz [67].

^b^ Rapeseed biodiesels A and B were prepared using 0.05% and 0.5% (w/w) KOH catalyst respectively.

Figures 8C and 8D show the ^1^H LF-NMR relaxation time distributions analyzed for samples A and B respectively, using the WinDXP ILT software package and PDCO. According to the WinDXP ILT relaxation time distributions, sample B consists of mainly a single biodiesel peak, whereas sample A exhibits two overlapping peaks consistent with an oil-biodiesel mixture previously shown in Figure 6A. A possible solution to increase the resolution and sensitivity of the method may be the application of *L*_*1*_ regularization via PDCO. As Figure 8C shows for the PDCO solution, one of the oil peaks can now be distinguished (intrinsic *T*_*2*_ at approximately 220 ms).

This suggests that ^1^H LF-NMR is a simple and rapid tool for estimating acylglycerol content in biodiesel. It should be stated, however, that based on Figure 8D, it is not yet clear whether the limit of detection of the proposed method will allow detecting low acylglycerol residues in biodiesels that don't meet international standards.

Several process and reaction monitoring applications, using ^1^H LF-NMR either spectroscopically or using relaxation analysis, have been described in the literature ([68-71] and references therein). More recently, Linck et al. [72] have shown the potential of applying a mobile LF ^1^H NMR spectrometer for the analysis and monitoring of biodiesel production. Cabeça et al. [73] suggested a method for off-line monitoring the biodiesel TE reaction using monoexponential fitting. In that study, monoexponential *T*_*2*_ measurements were used to track the progression of the reaction until completion. In the case, however, where residues of methanol remained in the sample, an increase in *T*_*2*_ value was observed. This is consistent with the previous result shown for a biodiesel-methanol mixture (Figure 6D). Likewise, glycerol residues would lead to a decrease in the calculated *T*_*2*_ value, even though the reaction may have been completed. Since monoexponential fitting does not provide information regarding individual constituents, rather an average estimation of all *T*_*2*_s in regards to their content in the sample, the application of relaxation time distribution analysis using PDCO is hereby described. Using this approach, both at-line and off-line process monitoring can be performed, by tracking the disappearance of the oil peak in relation to the biodiesel peaks.

**Figure 9:**
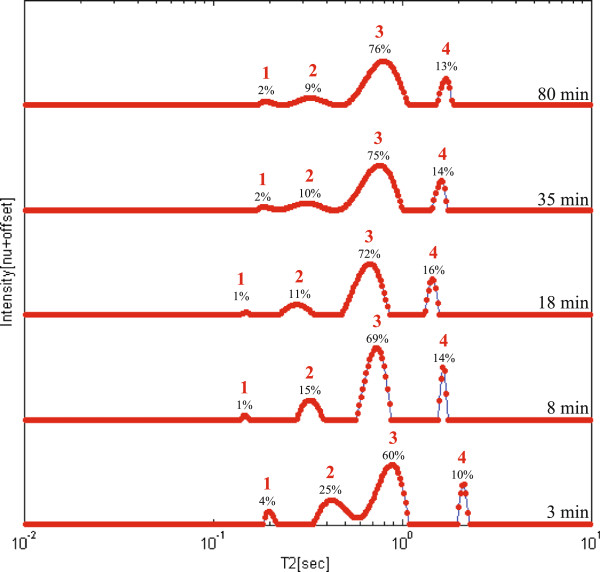
**At-line monitoring of the TE reaction using relaxation time distribution experiments.** Monitoring is shown using the combined 1D continuous distribution of exponentials of five samples acquired at different times in the course of the TE reaction of rapeseed oil into biodiesel. Samples were analyzed using PDCO. The content of each component, in relation to other components in the mixture, is shown on each relaxation time distribution.

At-line monitoring of the TE process of rapeseed oil was performed by collecting aliquots from the reaction flask, and immediately measuring them using relaxation time distribution, without further cleaning or tempering. Figure 9 shows the combined relaxation time distributions of five of the samples collected throughout the reaction, according to the order of collection, along with a rapeseed oil sample as reference. Since the TE reaction is performed at 50°C, and the ^1^H LF-NMR instrument operates at 40°C, there was a small influence to the relaxation time distributions, that can be observed from the shifts in the position of the peaks. Despite of this change, four peaks are clearly resolved for each sample.

According to previous peak assignment, peak 3 was the main biodiesel component and peak 4 was attributed to methanol. Peaks 1 and 2 were previously related to oil components. As can be seen, their relative intensities reach equilibrium but are not reduced to zero intensity as would have been expected. In order to assign these peaks, the sample collected following 110 min from the beginning of the reaction, was allowed to separate into two phases for twenty minutes, and then an aliquot from the upper layer was measured using relaxation time distribution. The analyzed distribution showed a result consistent with a biodiesel sample (without oil residues), as previously shown in Figure 7. It was therefore assumed that peak 2 belong both to glycerol and residues of oil components. The shift in the position of the glycerol component, to a higher *T*_*2*_ value is caused due to its solubility in methanol (relaxation time distribution of different methanol-glycerol mixtures is shown in Additional file 3). Peak 1 is more difficult to assign with certainty. At short reaction times it most probably originates from the oil component. However at longer periods this is not probable, especially since it disappears when measuring the upper layer alone. This remains for further study.

**Figure 10:**
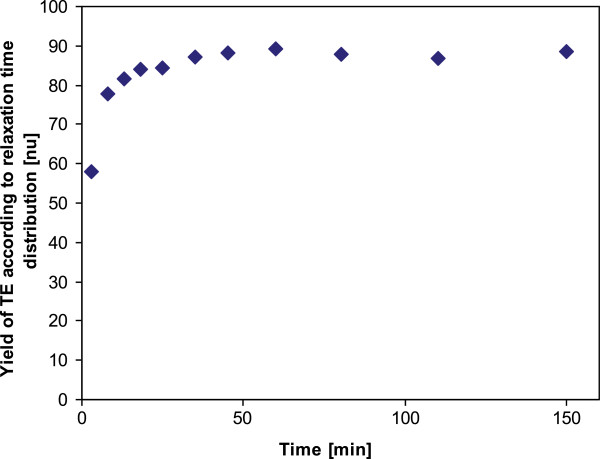
**The kinetics of rapeseed oil TE as calculated using relaxation time distribution.** Measurements were performed at-line using aliquots collected from the reaction flask. Samples were analyzed using PDCO.

The kinetics of the reaction according to the oil to biodiesel peaks area ((peak2/peak2+peak 3)*100%) is shown in Figure 10. As expected, the reaction progresses logarithmically, and a plateau is achieved at approximately 100 min. This trend is consistent with the results presented by Rashid and Anwar [74] for the kinetics of a rapeseed oil TE reaction at the same conditions. However, since the oil and glycerol peaks cannot be distinguished, the maximum calculated yield achieved is about 90%. This suggests that using the methodology described, the actual yield of the reaction cannot be accurately determined. Assessment of actual yields requires at least one separation step. This takes time, and therefore cannot be performed at-line.

To examine the possibility of calculating yields using relaxation time distribution, correlation of the yield calculated according to the relaxation time distribution (predictor) to the yield of TE calculated using ^1^H HR-NMR (predicted) was performed. For this purpose, six samples were collected while TE reaction of rapeseed oil was proceeding, and immediately placed on ice. Following phase separation, glycerol was drained and the biodiesel was cleaned. Three additional samples prepared using low catalyst concentration (0.05%, 0.1% and 0.15% w/w KOH) were also used in the correlation, in order to broaden the range of yields. Since the prepared samples were cleaned, no glycerol or methanol residues were shown in the relaxation time distributions. The yield based on relaxation time distribution was therefore calculated from the oil to biodiesel peaks area.

This correlation yielded an excellent linear fit (*y* = 0.78*x* + 20.50, *R*^2^ = 0.99; the Figure was added in an additional file [see Additional file 4]). This suggests that the proposed method is a good tool for monitoring and calculating the yield of the TE reaction. Its disadvantage, however, is the time consuming cleaning process which makes this an off-line tool.

A combined tool is therefore suggested, were monitoring of the TE reaction is qualitatively performed at-line, until reaching equilibrium, and yield quantification is carried out off-line, following phase separation and cleaning of the upper layer.

#### Monitoring of the in situ TE process of pomace

Reutilization of bio-wastes as alternative energy holds the possibility of reducing their impact on the environment, along with the potential to expand the currently limited biodiesel industry [10]. Solid olive mill waste is a promising composite feedstock for the biofuel industry because of its high content of both lipids and cellulosic materials. In this study, relaxation time distribution experiments were performed to monitor the TE reaction of the in situ conversion of oil in olive pomace to biodiesel.

Olive mill pomace contains low grade oil with high content of FFAs (>3%). This leads to the formation of soaps in a base catalyzed reaction, which causes an increase in viscosity or formation of gels that interfere in the reaction as well as with the separation of glycerol [65]. In order to carry out the in situ TE reaction, a preliminary acidic esterification step is required in which FFAs are transformed into FAMEs. If carried out for long periods, oil is additionally transesterified into biodiesel. This is energy consuming because it is performed at a relatively high temperature (100°C), and thus not desirable. In the current study, the in situ process consisted of an acidic esterification step, followed by alkali TE. As a final step, the solid pomace with methoxide sludge was washed with n-hexane to remove oil and FAME residues in order to achieve low oil content pomace.

**Figure 11:**
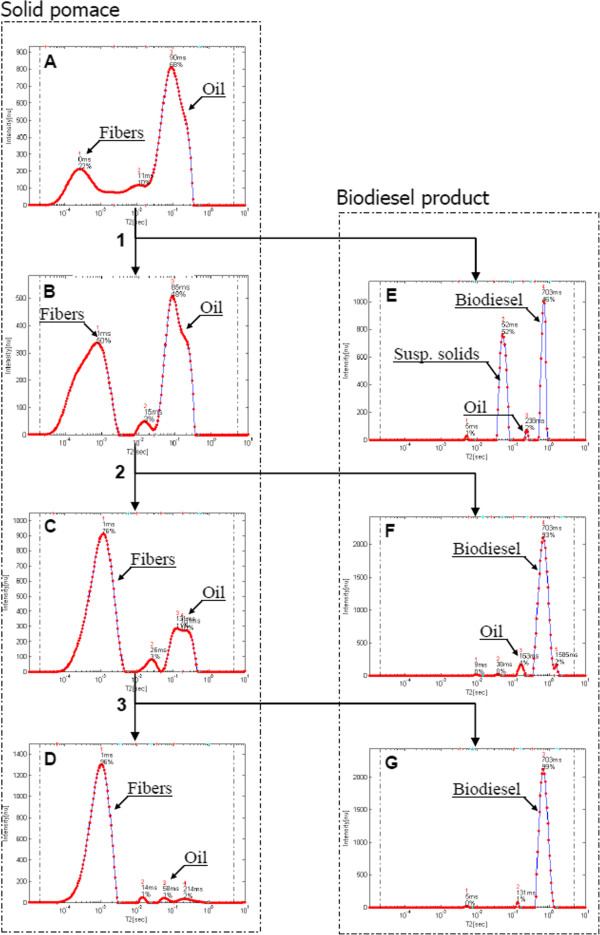
**Monitoring of the in-situ TE process of pomace.** Samples were collected following (1) acid esterification (2) alkali TE, and (3) n-hexane extraction using relaxation time distributions. Figures are ordered according to the appropriate step in the reaction process. (**A**)-(**D**) show the results for the solid pomace, and (**E**)-(**G**) for the biodiesel product.

Monitoring of this process is shown in Figures 11A-11D and 11E-11G for the analyzed relaxation time distributions of the solid pomace and biodiesel products respectively, using the WinDXP ILT toolbox. Data was acquired at each step of the reaction. 1, 2, and 3 mark each of the reaction steps: acid esterification, alkali TE and n-hexane extraction.

Based on the previous peaks assignment in olives, the main constituents in the initial dry solid pomace sample consisted of oil (intrinsic peak at approximately 90 ms) and fibers (intrinsic peak at approximately 0.1 ms) components, as shown on Figure 11A. Following the esterification step, the area of these two types of components was changed, meaning the oil component was reduced while the fibers increased (Figure 11B). This suggested that not only FFAs were reacted following this step, but also oil was transesterified into FAMEs. This was assessed by analyzing the liquid product following this step (Figure 11E). Here a biodiesel peak was observed. In addition, another major component was detected, which was attributed to suspended solids in the sample because the liquid fraction was opaque in color. The increase in the fibers peak area was attributed to the formation of more mobile constituents, following breaking down of fibers which could not be detected before because of their fast relaxation. As expected, an additional reduction in the relative content of the oil fraction was accomplished following the alkali TE step (Figure 11C). Still additional optimization of these process parameters is required because the reacted pomace contained substantial amounts of oil. The residual oil was successfully extracted using n-hexane as shown on Figure 11D. Monitoring of the liquid fraction following each step confirmed the successful production of biodiesel, and showed the presence of impurities in the product (Figures 11E-11G).

The achieved low oil content pomace, which is very rich in fibrous material, can be further used for production of bioethanol. In a collaborative work with Dr. Ely Morag (Designers Energy Ltd, Israel) and Prof. Edward A. Bayer (Weizmann Institute of Science, Israel), it was found that reducing the oil content in olive pomace, prior to cellulase enzymatic hydrolysis, showed significant increase of sugar release from the pomace cellulosic fraction. In addition, the acidic and alkali treatments described above for the in situ biodiesel production significantly supported the enzymatic pre-treating procedure. This suggests that relaxation time distribution experiments can be applied for monitoring the production of other biofuels, including bioethanol. Such a study is currently being carried out.

In this work, monitoring of the in situ biodiesel TE process was performed qualitatively. However, development of calibration curves or the use of internal standards can reveal important quantitative information, including oil content, conversion yield, quantity of biodiesel produced, and more. These applications are currently being developed by our team for optimization of the in situ TE process of olive pomace.

### Quality assessment of the biodiesel product

As previously shown, relaxation time distribution experiments can be applied to calculate the yield of the TE reaction. This gives an approximation of residual acylglycerol content in the biodiesel, which is a qualitative indication of whether it meets international standards. Another qualitative aspect is the biodiesel FA composition, which affects several physical properties of the fuel. Several of the parameters specified in international biodiesel standards are determined directly by choice of feedstock, according to their FA profile. Prestes et al. [36] showed that it is possible to predict several of these parameters by measuring transverse relaxation data of oilseeds, even without extracting and transesterifying the oil. However, evaluation of physical properties of the biodiesel product is also of paramount importance, especially for biodiesel and/or petrodiesel blends.

In this study, transverse relaxation data of six different types of biodiesels and nine petrodiesel-biodiesel mixture samples (BXX, where XX stands for the amount of castor biodiesel in the mixture) was acquired using LF-NMR. Hierarchical Cluster Analysis (HCA) was applied to explore the relative distance and grouping of samples according to the pre-processed relaxation data. Biodiesel viscosities were calculated from their FA composition, as suggested by Allen et al. [13].

Biodiesel consists of a mixture of FAMEs in which each constituent contributes to the overall viscosity. Viscosity increases with chain length and with increasing degree of saturation [75]. The main FAMEs and calculated viscosities are shown on Table 3 (observations are ordered by ascending calculated viscosities). Even though the viscosity of the BXX samples cannot be determined by GC, samples were assigned to viscosity groups based on the HCA dendrogram mixture.

**Table 3:** FAME profiles according to GC and calculated viscosities of the six biodiesels produced

**Sample**^**a**^	**FAME composition**^**b**^**[%]**									**Calculated viscosities [mm**^**2**^**/s]**
	**C**_**16:0**_	**C**_**18:0**_	**C**_**18:1**_	**C**_**18:2**_	**C**_**18:3**_	**C**_**20:0**_	**C**_**20:1**_	**C**_**22:1**_	**C**_**18:1-OH**_	
Linseed	5.6	4.6	18.2	15.4	56.1	-	-	-	-	3.60
Soy	10.8	3.6	25.6	53.4	5.5	0.4	0.4	0.4	-	3.99
Canola	4.8	1.6	63.7	20.3	7.2	0.6	1.3	-	-	4.25
Olive	10.6	2.8	77.3	7.4	0.6	0.4	-	-	-	4.45
Mustard	1.7	1.0	9.8	13.8	12.9	0.8	6.2	51.4	-	5.52
Castor	1.0	1.1	3.0	3.9	-	-	-	-	91.1	13.76

^a^ Samples are ordered by ascending calculated viscosities.

^b^ Only major FAMEs are shown.

**Figure 12:**
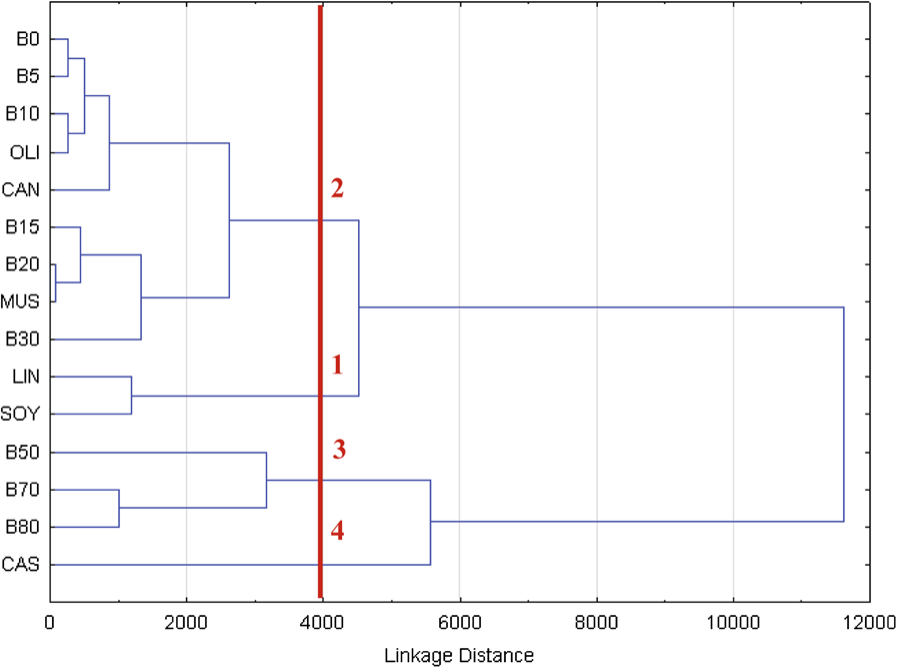
**Viscosity assessment of the biodiesel product using chemometrics.** HCA dendrogram of six biodiesels (LIN: linseed, SOY: soy, CAN: canola, OLI: olive, MUS: mustard and CAS: castor) and nine petrodiesel-biodiesel mixture samples (BXX, where XX stands for the amount of castor biodiesel in the mixture) was calculated using the acquired transverse relaxation signals. A linkage distance threshold of 4000 yielded four groups which were assigned to different viscosities.

Figure 12 shows the HCA dendrogram. Using a linkage distance threshold of 4000, four distinct groups (marked 1–4) were observed, that can be related to the viscosity of the samples. Linseed and soy biodiesels, rich in the polyunsaturated, linoleic and linolenic acids, showed the lowest viscosities (3.60 and 3.99 mm^2^/s respectively) and were thus assigned to group 1. Group 2 was subdivided into two groups. The first included the olive and canola samples (rich in the monounsaturated oleic acid, 4.25 and 4.45 mm^2^/s respectively) and B0-B10 mixtures. The second included the mustard sample (rich in erucic acid, 5.52 mm^2^/s) and B15-B30 mixtures. Erucic acid increases viscosity because of its larger chain length compared to the more common 18 carbon components. Group 3 comprised of only mixture samples (B50-B80). Even though their exact viscosity values are unknown, based on the mixing ratio they should have higher viscosity than B30 and lower than B100 (pure castor biodiesel). Therefore, the highest viscosity group 4 was assigned to castor biodiesel, having very high viscosity (13.76 mm^2^/s) imparted by intermolecular hydrogen bonds caused by ricinoleic acid.

The proposed methodology allows the assignment into viscosity groups of samples whose viscosity is unknown, simply by acquiring their transverse ^1^H LF-NMR signals and analyzing them using chemometric tools like HCA and PCA. These methods can also be applied to find biodiesel samples that have residues of unreacted acylglycerol and/or methanol, and poorly separated and cleaned glycerol and water, provided a high quality biodiesel of the same source is used for comparison. All the aforementioned residues influence the viscosity of the sample, and accordingly affect the acquired transverse relaxation signal.

**Figure 13:**
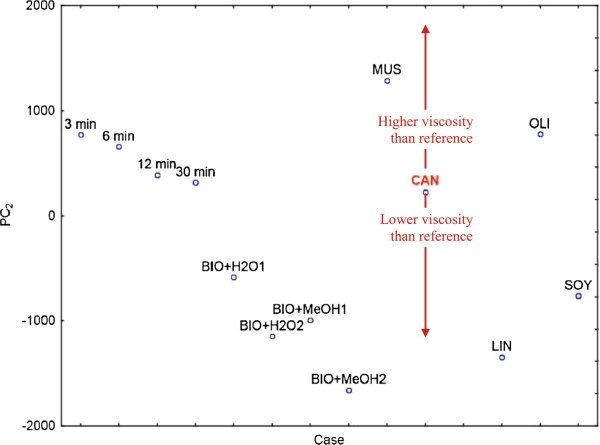
**Identification of unwanted residues in biodiesel samples using chemometrics.** Transverse relaxation data of the 6 biodiesels; samples collected throughout the monitoring experiment at 3, 6, 12 and 30 min; and mixture samples of rapeseed biodiesel with water or methanol (BIO+H2O1/2 and BIO+MeOH1/2 consist of 3.33% and 6.66% v/v water or methanol in rapeseed biodiesel respectively) are shown in a *PC*_*2*_ score line plot. The *PC*_*2*_ scale of the plot was enlarged, in order to focus on the area of interest, therefore the castor biodiesel sample is not shown. The rapeseed biodiesel is marked to easily estimate biodiesel samples with higher or lower viscosities.

An example is shown on the *PC*_*2*_ score line plot calculated using PCA on the pre-processed transverse relaxation signals (Figure 13). In order to show the effect of residues on the position of samples in the score line plot, the following samples were used along with all the biodiesels described before: (a) four of the samples collected and used to calculate the yield of TE reaction (3, 6, 12 and 30 min), to demonstrate acylglycerol residues; (b) two biodiesel samples with low water content (BIO+H2O1 and BIO+H2O2 consist of 3.33% and 6.66% v/v water in rapeseed biodiesel respectively); and (c) two biodiesel samples with low methanol content (BIO+MeOH1 and BIO+MeOH2 consist of 3.33% and 6.66% v/v methanol in rapeseed biodiesel respectively). The *PC*_*2*_ scale of the plot was enlarged, in order to focus on the area of interest, therefore the castor biodiesel sample is not shown. Following the established correlation of *PC*_*2*_ and viscosity [59], it is clearly demonstrated, that residues of water and/or methanol lead to lower than expected biodiesel viscosities, whereas high acylglycerol content lead to the opposite effect.

This tool allows to rapidly identify lower or higher viscosity samples than the one expected. More precisely, when using additionally samples whose viscosity is known (or estimated by their FAME profiles); it is possible to also approximate the viscosity value of the unknown samples, rather than simply assign them as higher or lower viscosities than the reference.

## Conclusions

The novel ^1^H LF-NMR applications presented offer great potential to the biodiesel industry for characterizing new alternative biodiesel resources in their whole conformation, monitoring the biodiesel TE reaction and evaluating the quality of the final product. In addition, the new integrated computational-experimental approach for ^1^H LF-NMR relaxometry presented, suggests better resolved relaxation time distributions and more accurate solutions.

## Materials and methods

### Materials

CDCl_3_ (99.8% + 0.05% v/v TMS) was purchased from D-Chem Ltd., Israel. Glycerol was purchased in a local pharmacy (Ph Eur grade, Floris, Israel). All other chemicals and reagents used in this study were analytical grade. All oils were purchased from local suppliers. Biodiesels were prepared through a base catalyzed reaction using several of the purchased oils. Petrodiesel was purchased at a local gas station.

Fresh olive fruits were harvested from a plot located in the central Negev, Israel. The whole dry olive sample was oven dried at 70°C for 72 h to remove excess moisture. Olive mill pomace was collected from Darawsha olive press (Iksal, Israel).

### ^1^H LF-NMR signal acquisition

Relaxation time distribution experiments for peaks assignment of the olive oil and olive stone samples were performed on a DRX23 bench-top pulsed NMR analyzer (Resonance Instruments, Witney, UK) operating at 25°C, equipped with a permanent magnet and a 10 mm probe head operating at 23.4 MHz. Prior to measurement, samples were equilibrated at 25°C for 1 h. All other ^1^H LF-NMR measurements were performed on a Maran bench-top pulsed NMR analyzer (Resonance Instruments, Witney, UK) operating at 40°C, equipped with a permanent magnet and different diameter probe heads, operating at 23.4 MHz. Prior to measurement, samples were equilibrated at 40°C for 1 h.

1D relaxometry experiments were performed using a CPMG pulse sequence. This multiple sequence consists of applying a single 90°; pulse followed by multiple consecutive 180° pulses. This allows measuring transverse relaxation, which results from spin-spin interactions.

2D cross-correlation experiments were performed by a *T*_*1*_*T*_*2*_ sequence, where an inversion recovery step [180°–*t*_*1*_ is inserted in front of the CPMG sequence [16]. Here the *T*_*1*_ dimension is acquired by repeating the sequence a determined number of steps, where the space *t*_*1*_ is varied logarithmically between runs. The *t*_*1*_ period is dominated by longitudinal relaxation, including possible longitudinal cross relaxation processes; while the *t*_*2*_ period is dominated by transverse relaxation processes [15].

All relaxation time distribution experiments on solid and liquid samples, were analyzed without further preparation. Samples were inserted in glass NMR tubes as whole, without using solvents or further crushing. Parameters in each pulse sequence were tailored according to the type of experiment and material. Following data acquisition the signal was phase rotated and only the main channel was used for the analyses.

### Data analysis

#### Chemometrics

In this work, chemometric data analysis tools included PCA for unsupervised data exploration and PLS for creating multidimensional regression curve fitting [76-78]. Both these methods extract PCs and loadings to maximize the original variance and reduce dimensionality; hence they describe the data in a more condensed form. The PCs are mutually orthogonal and their extraction is such that the first PC holds the maximum variance, the second holds the second-maximum variance, and so on.

HCA was applied for clustering. HCA is an unsupervised clustering technique that examines the interpoint distances between all samples in row space and represents them in a dendrogram. To generate the dendrogram, a common approach is to initially treat each sample as a cluster and join closest clusters together. The process is repeated until only one group remains [77]. The amalgamation rule used was complete linkage and distances between clusters were calculated according to Euclidean distances. Analysis of results was carried out using the distance dendrogram.

All chemometric methods were computed using STATISTICA software (ver. 11.0, StatSoft). PCA and PLS were applied using the NIPALS algorithm on the covariance matrices. Transverse relaxation data was either used as acquired, or pre-processed by dividing the entire signal of each sample by its first (and highest) intensity.

Goodness of fit for the PLS regression model was determined using the validation set. RMSEP was calculated using Eq. (1):

(1) $$\mathrm{RMSEP}=\sqrt{\frac{\overset{n}{\underset{i=1}{\sum }}{\left(x_{i,\mathrm{predicted}}-x_{i,\mathrm{calculated}}\right)}^{2}}{n}}$$

where *n* is the number of observations.

#### Monoexponential and continuous distribution fitting of 1D relaxation signals

Monoexponential fitting was performed with the WinFit software package (Oxford Instruments, UK).

Conversion of the relaxation signal into a continuous distribution of relaxation components is performed using Eq. (2), where the probability density *f*(*T*_*2*_) is calculated applying ILT, *s*(*t*) is the relaxation signal acquired with ^1^H LF-NMR at time *t*, *T*_*2*_ are the time constants, and *E*(*t*) is the measurements error:

(2) $$s\left(t\right)=\int e^{-t/T_{2}}f\left(T_{2}\right)dT_{2}+E\left(t\right).$$

The most common numerical method implemented today for dealing with ill-posed problems of this kind is based on *L*_*2*_-norm regularization, where Eq. (2) is approximated by a discretized matrix form, and minimized according to the *L*_*2*_-norm expression:

(3) $$\begin{array}{l} f=\mathrm{argmin}\left\|s-{\left(\mathrm{Kf}\right\|}_{2}^{2}\right)+\lambda {\left\|f\right\|}_{{}_{2}}^{2},\\ f\geq 0 \end{array}$$

where *K* is the discrete Laplace transform, and *λ* is the *L*_*2*_ weight. This type of regularization, however, can significantly distort the solution by contributing to the broadening of peaks.

In this work, we applied a novel numerical optimization method developed by our research team for analyzing the ^1^H LF-NMR relaxometry data. Full description of the algorithm is described in an article recently (2012) submitted to Concepts in Magnetic Resonance A Journal. The new method applies the PDCO solver that can be adjusted to solve the inverse problem with nonnegativity constraints and an *L*_*1*_ regularization term that stabilizes the solution process without introducing the typical *L*_*2*_ peak broadening. The underlying principle is that all structured signals have sparse representation in an appropriate coordinate system, and using such a system/dictionary typically results in better solutions with a relatively low level of noise.

The mathematical formulation of our proposed method is the linearly constrained convex optimization problem

(4) $$\begin{array}{l} \min_{f,c,r}\lambda_{1}{\left\|c\right\|}_{1}+\frac{1}{2}\lambda_{2}{\left\|c\right\|}_{2}^{2}+\frac{1}{2}{\left\|r\right\|}_{2}^{2}\\ s.t.\;\mathrm{Kf}+r=s,\\ \;-f+\mathrm{Bc}=0,\;f\geq 0, \end{array}$$

where *K* is the discrete Laplace transform, *f* is the unknown spectrum vector, *s* is the measurements vector, *r* is the residual vector, and *B* is a sparsifying dictionary.

*L*_*2*_ calculations of relaxation time distribution were de-convoluted as a continuous distribution of relaxation times with the WinDXP ILT software package (Distributed ExPonential Analysis, Oxford Instruments, UK).

#### Continuous distribution fitting of 2D cross correlation relaxometry

2D cross correlation relaxometry signals were analyzed by 2D-ILT as described in [16], using an in-house program written in MATLAB as detailed by Hills et al. [15]. Briefly, the acquired 2D array of CPMG echo trains *s*(*t*_*1*_*t*_*2*_) is given as

(5) $$\begin{array}{c} s\left(t_{1},t_{2}\right)=\iint \left(1-2e^{-t_{1}/T_{1}}\right)e^{-t_{2}/T_{2}}f\left(T_{1},T_{2}\right)dT_{1}dT_{2}\\ \;+E\left(t_{1},t_{2}\right), \end{array}$$

where *s*(*t*_*1*_*t*_*2*_) is the relaxation signal acquired at *t*_*1*_ and *t*_*2*_ times, and the function *f*(*T*_*1*_*T*_*2*_) corresponds to the probability density of molecules with relaxation times of *T*_*1*_, *T*_*2*_.

### Extraction

#### Oil extraction

For non-quantitative oil extraction, oil-containing materials were crushed manually in a mortar and incubated overnight with n-hexane in an orbital shaker at 25°C. The oil and n-hexane solution was then evaporated under a mild vacuum with a rotary evaporator. In cases where large volumes of oil were required (>10 ml), a cold-pressed extruder was used (Komet CA 59G, Monchengladbach, Germany).

Oil extraction for oil content measurements was carried out using a modified procedure according to the AOCS official method [79], using a soxhlet apparatus and n-hexane. Briefly, about 2 g of oil containing materials were manually crushed with a mortar and positioned in the extraction chamber for 24 h. The oil and n-hexane mixture was then evaporated under mild vacuum to obtain pure oil.

#### Alkali TE

Prior to biodiesel production, oils were heated to 80°C for 1 h to evaporate water, and then allowed to cool down to room temperature. Biodiesels were prepared in a batch laboratory scale TE process with methanol and KOH. Briefly, a potassium hydroxide solution was freshly prepared by mixing methanol (1:6 oil to methanol mol/mol) with KOH (100 g kg^-1^ of the oil). The reaction was carried out for 1 h under reflux at 50°C with constant stirring and then allowed to cool down to room temperature. The mixture was then transferred to a separating funnel and allowed to stand for approximately 1 h. The bottom layer (glycerol, methanol and most of the catalyst) was drained out. The upper layer (FAMEs, some methanol and traces of the catalyst) was cleaned thoroughly by washing 5 times with warm (~50°C) de-ionized water. The solution was then heated to 80°C for 30 min until cleared.

#### In situ TE of olive mill waste pomace

Prior to in situ TE reaction samples were oven dried at 70°C for 72 h to remove excess moisture. The dry pomace was then ground using a Hsiangtai electric grinder (Taipei Hsien, Taiwan) to provide more surface area for the reaction.

Because of high FFA content (2-13% FFA according to a titration procedure), pomace was first esterified using H_2_SO_4_. Briefly, 100 g of dry and grounded pomace were reacted under reflux and stirring, with 2 ml H_2_SO_4_ (0.094 M) and 400 ml methanol. The reaction was carried out for 1 h at 65°C. An alkali TE reaction was then performed as described before. Prior to reaction, the remaining sulfuric acid was neutralized using a KOH in methanol solution (4.8% w/w). Eventually, 200 ml n-hexane was added to extract the biodiesel. The final product was separated into solid and liquid fractions by filtration. The liquid fraction was transferred to a separating funnel and allowed to settle for 30 min. The bottom layer consisting of methanol, residual catalyst and soluble sub-millimeter particles was removed, and the upper layer consisting of the biodiesel produced and n-hexane was washed several times with warm (~50°C) de-ionized water. The biodiesel produced was eventually recovered using a rotary evaporator, and heated to 80°C for 60 min until cleared.

### Identification

#### GC

GC was used for identification and quantification of FAMEs. The FA composition was used to calculate the kinematic viscosity of biodiesels as described by Allen et al. [13] using Eq. (6). *μ*_*i*_ and *μ*_*0*_ are the kinematic viscosities at 40°C of each FA and biodiesel, respectively; *y*_*i*_ is the mass fraction of each FA. Neat FA viscosities, given as FAMEs, were taken from [75]. The viscosity of arachidic acid (20:0) was assumed to be similar to that of stearic acid (18:0), as suggested by Allen et al. [13].

(6) $$\ln\mu_{0}=\sum_{i=1}^{n}y_{i}\;\ln\;\mu_{i}$$

GC analyses were conducted on a Varian 3400 apparatus (Palo Alto, CA, USA) equipped with a flame ionization detector and a Stabilwax-DA capillary column (RESTEK, Bellefonte, US; Dimensions: 15 m × 0.32 mm × 0.25 μm).

#### ^1^H HR-NMR

^1^H HR-NMR analyses were conducted on a Bruker DMX-500 NMR spectrometer (Bruker, Germany) operating at 500 MHz. ^1^H HR-NMR was used to monitor acylglycerol residues in biodiesel. Prior to measurement, samples were dissolved in CDCl_3_.

The yield (*C*) of TE reaction was calculated according to Eq. (7), where *A*_*ME*_ is the integrated signal at 3.7 ppm corresponding to methyl protons in methyl esters; and *A*_*CH2*_ is the integrated signal at 2.3 ppm due to methylene protons adjacent to the ester group in triglycerides [65].

(7) $$C=100*\frac{2A_{\mathrm{ME}}}{3A_{CH_{2}}}$$

## Abbreviations

1D: One dimensional; 2D: Two dimensional; CP: Cross peak; CPMG: Carr, Purcell, Meiboom and Gill; FA: Fatty acid; FFA: Free fatty acid; GC: Gas chromatograph; HCA: Hierarchal cluster analysis; HR-NMR: High resolution nuclear magnetic resonance; ILT: Inverse laplace transform; LF-NMR: Low field nuclear magnetic resonance; PC: Principal component; PCA: Principal component analysis; PDCO: Primal-dual interior method for convex objectives; PLS: Partial least squares; RMSEP: Root mean squared error of prediction; TE: Transesterification.

## Competing interests

The authors declare that they have no competing interests.

## Authors’ contributions

PB designed and performed most of the experiments, analyzed results, contributed in the establishment and validation of the new PDCO algorithm, and drafted the manuscript. AL performed the monitoring of the TE process and calculation of yield using relaxation time distribution experiments. OE performed and optimized the monitoring of the in situ TE process of pomace using relaxation time distribution experiments. OL, YP and MS formulated, designed and established the PDCO algorithm. ZW led and coordinated the overall project, contributed to the development of the experimental design and proofread the manuscript. All authors read and approved the final manuscript.

## Supplementary Material

Additional file 1 **Correlation of the slope and T**_**2 **_**of the 9 different oils.** Slopes were calculated by fitting the extracted *PC*_*1*_ and *PC*_*2*_ of each of the individual oils separately. *T*_2_ was the average value for each of the 4 samples per oil, calculated using monoexponential fitting. Click here for file

Additional file 2 **Correlation of measured *vs. *calculated oil content of the nine different oils through PLS.** The correlation was performed on the validation set. Click here for file

Additional file 3 **Relaxation time distribution of different methanol-glycerol mixtures.** The methanol to glycerol ratio (M:G) of each mixture is shown on each plot. The relaxation time distribution of methanol (MeOH) and glycerol (Gly) are shown for reference. Click here for file

Additional file 4 **Correlation of the yield calculated from **^**1**^**H LF-NMR to that of **^**1**^**H HR-NMR.** The yield based on relaxation time distribution was calculated from the oil to biodiesel peaks area. The samples used for the correlation include six samples collected while TE reaction of rapeseed oil was proceeding, following separation and cleaning, and three additional samples prepared using low catalyst concentration (0.05%, 0.1% and 0.15% w/w KOH). Click here for file
