# Supplementary figures and images for: Novel 1H low field nuclear magnetic resonance applications for the field of biodiesel
Source: Biotechnol Biofuels. 2013 Apr 16;6:55. doi: 10.1186/1754-6834-6-55 (PMC3689644; doi:10.1186/1754-6834-6-55)

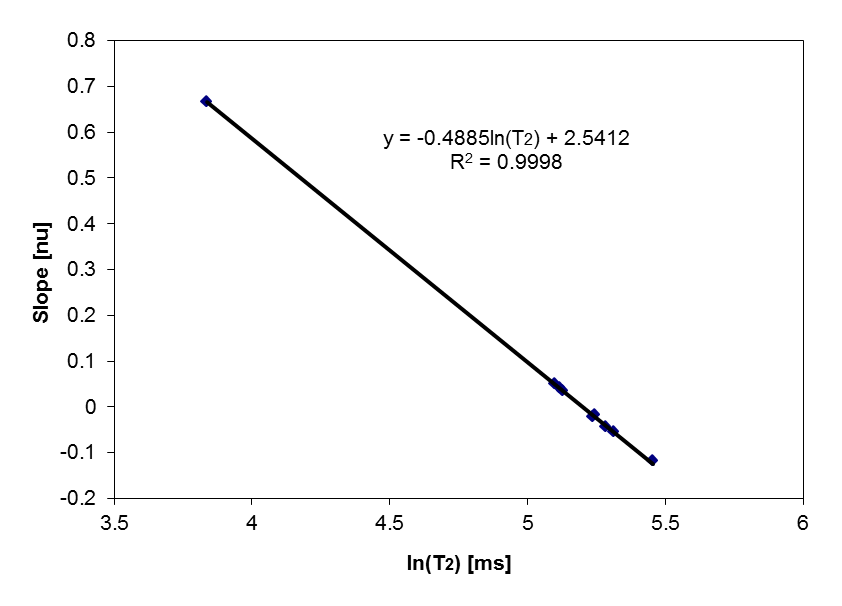

Supplement: Additional file 1 — Correlation of the slope and T2 of the 9 different oils. Slopes were calculated by fitting the extracted PC1 and PC2 of each of the individual oils separately. T2 was the average value for each of the 4 samples per oil, calculated using monoexponential fitting. [file 1754-6834-6-55-S1.doc]

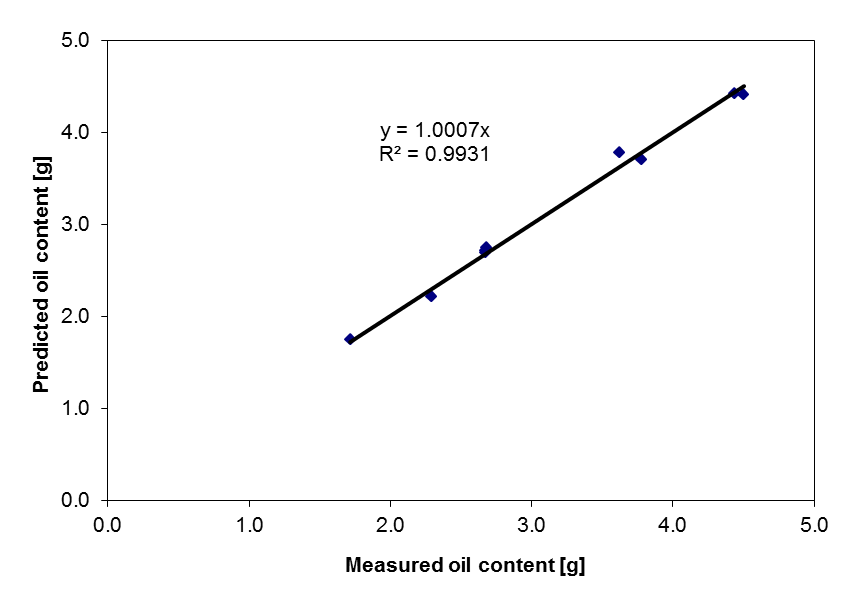

Supplement: Additional file 2 — Correlation of measured vs. calculated oil content of the nine different oils through PLS. The correlation was performed on the validation set. [file 1754-6834-6-55-S2.doc]

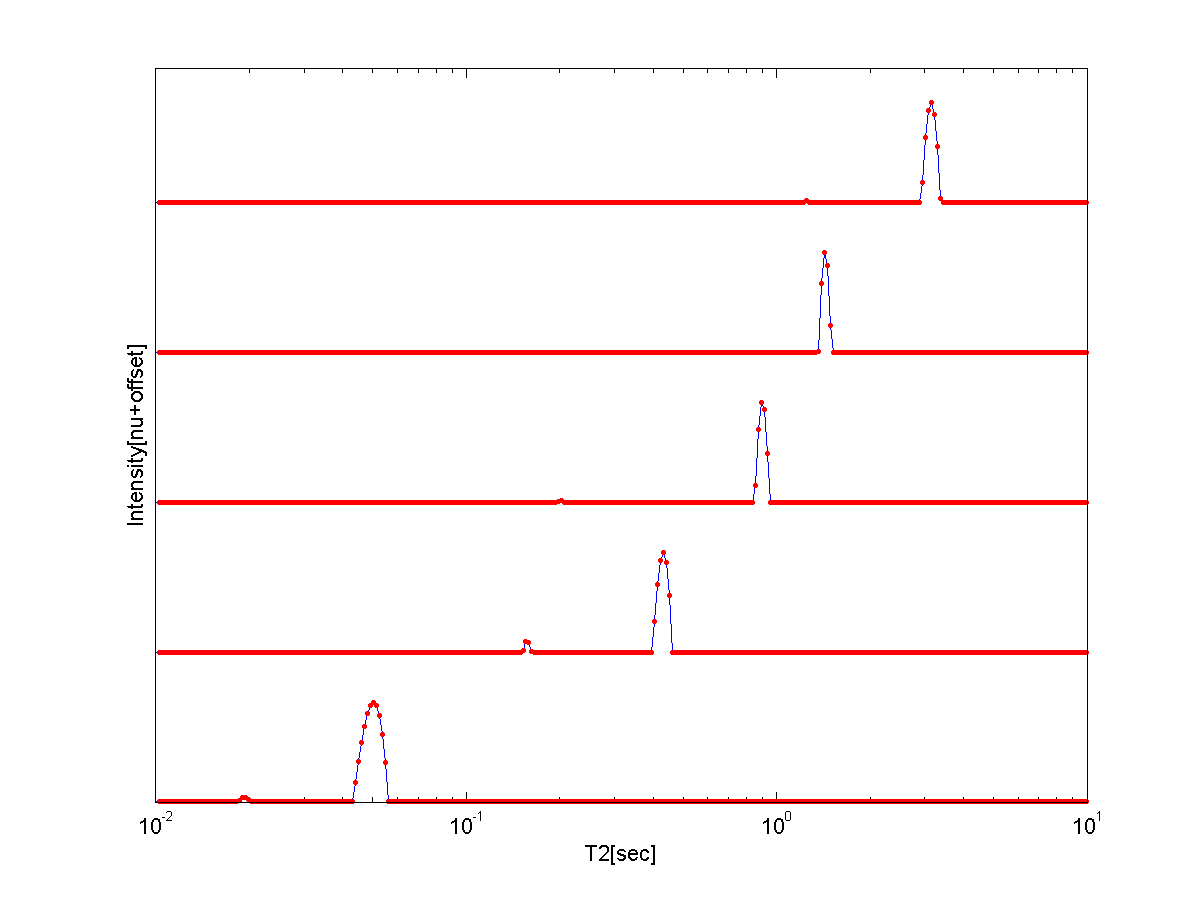


MeOH

M:G , 2:1

M:G , 1:1

M:G , 1:2

Gly

Supplement: Additional file 3 — Relaxation time distribution of different methanol-glycerol mixtures. The methanol to glycerol ratio (M:G) of each mixture is shown on each plot. The relaxation time distribution of methanol (MeOH) and glycerol (Gly) are shown for reference. [file 1754-6834-6-55-S3.doc]

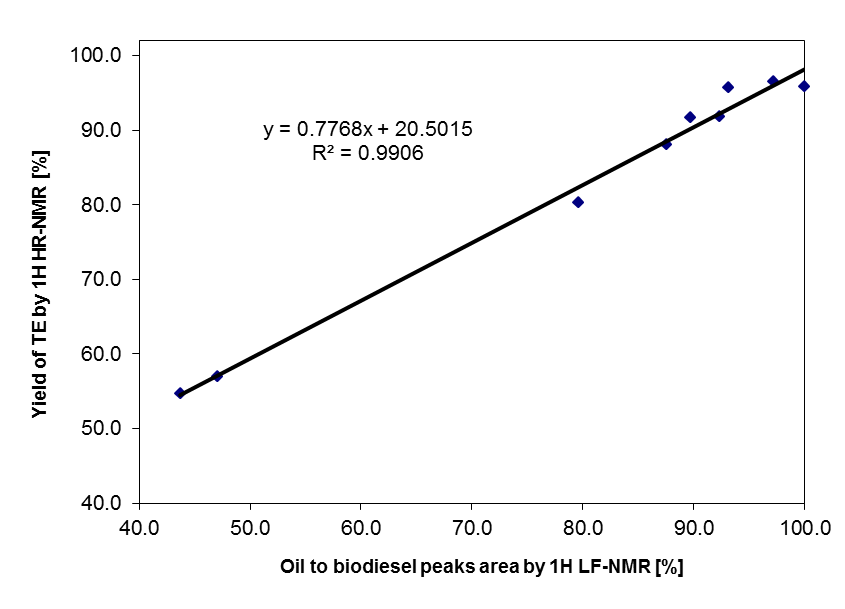

Supplement: Additional file 4 — Correlation of the yield calculated from 1H LF-NMR to that of 1H HR-NMR. The yield based on relaxation time distribution was calculated from the oil to biodiesel peaks area. The samples used for the correlation include six samples collected while TE reaction of rapeseed oil was proceeding, following separation and cleaning, and three additional samples prepared using low catalyst concentration (0.05%, 0.1% and 0.15% w/w KOH). [file 1754-6834-6-55-S4.doc]
